# Supplementary material for: Are carbon emissions trading and green financial instruments synergistic? -Comprehensive quantitative research based on content analysis
Source: PLoS One. 2024 Mar 7;19(3):e0298601. doi: 10.1371/journal.pone.0298601 (PMC10919601; doi:10.1371/journal.pone.0298601)
Supplement: S1 Data — (ZIP) [file pone.0298601.s001.zip › Data/地级市Z_F工作报告.zip!/╦─┤¿/┼╩╓a╗¿╩╨/└┤╫╘╣1⁄2╓┌║┼í╛┬φ┐╦╩2╛▌═°í┐_2016.docx]

各位代表：

　　我代表市人民政府，向大会报告工作，请予审查，并请政协委员和其他列席会议的同志提出意见。

　　一、回顾“十二五”，我市建设发展取得新成绩

　　“十二五”时期，我国经济发展进入新常态，经济呈现持续下行的复杂态势。面对严峻挑战，在市委坚强领导下，在市人大、市政协监督支持下，市政府团结带领全市各族人民，迎难而上，开拓进取，坚定不移推进“三个加快建设”“三个走在全省前列”，坚持不懈打好“六大硬仗”，基本完成“十二五”规划主要目标。

　　——综合实力不断增强。与“十一五”末相比，地区生产总值由523.99亿元增加到925.18亿元，年均增长11.5%，人均GDP突破7万元；规模以上工业增加值年均增长14.1%；社会消费品零售总额由143.44亿元增加到286.20亿元，年均增长14.8%；一般公共预算收入由38.78亿元增加到53.34亿元，年均增长8.3%；全社会固定资产投资累计完成2654亿元，是“十一五”时期的2.51倍。按统计新口径初步核算，城镇居民人均可支配收入由17286元增加到30362元，年均增长12.1%；农村居民人均可支配收入由6757元增加到12861元，年均增长13.9%。

　　——城市品牌初步塑造。加快转变发展方式，大力推动产业升级、城市转型，中国钒钛之都、中国阳光花城、四川南向开放门户、国家现代农业示范区、中国阳光康养旅游城市成为彰显我市新形象、体现发展新内涵的重要品牌。建成国内规模最大的钒钛原料基地和潜力最大的钒钛低（微）合金钢机械深加工产业基地，成功举办中国（攀枝花）钒钛产业博览会，钒钛及相关产业产值占工业总产值比重大幅提升，初步实现了工业结构以钢铁为主向钢铁钒钛并进的战略转变。启动实施“五创联动”，成功创建为省级森林城市，顺利通过省级园林城市复审，加快构建区域性交通枢纽和商贸物流中心，花城景观打造卓有成效，环境空气质量优良率连续5年保持在90%以上，初步实现了从生产型工矿城市向综合型宜居宜业宜游城市的重大转型。大力发展现代农业，探索出了一条“以体系为支撑、向特色要效益”的山区特色高效农业发展道路，成为全国以整建制形式跻身第二批国家现代农业示范区的两个城市之一，是全省唯一的高效节水农业试点市，初步实现了从传统农业向现代特色农业的有效提升。在全国创造性提出中国阳光康养旅游城市概念并持之以恒推动建设，圆满举办首届中国康养产业发展论坛，“阳光花城·康养胜地”品牌成功打响，初步实现了从单一型旅游向康养旅游融合发展的创新突破。

　　——发展空间有序拓展。修编完成《攀枝花市城市总体规划（2011—2030）》。深入贯彻全域规划理念，成功打造红格、白马等特色小城镇，新型城镇化建设走在全省前列。统筹实施旧城改造，全面启动并有序推进新区建设，现代化新型城区初具规模，城镇化率由60.1%提高到64%以上，建成区面积由54.6平方公里扩大到74平方公里。加快建设城乡路网、电网、通信网，国省干线三年攻坚全面完成，炳仁路后段、龙密路、炳二区主干道、新密地大桥等建成通车，所有行政村全部通公路，电力主网结构更加完善，建成全国首个全光网城市。

　　——转型活力持续激发。稳步推进国资国企改革，组建国投、建设两大集团，市属经营性国有企业总资产较“十一五”末增加182%。市商行跨区域发展，市农商行组建运营，县域及三农金融服务实现全覆盖。深化行政审批制度改革，行政审批事项由434项减少到228项，非行政许可审批事项全部取消。资源税改革、矿山复垦等纳入全国试点，交通运输综合行政执法体制改革试点经验在全国推广。不断完善科技创新体系，建成一批公共创新服务平台，万人发明专利拥有量位居全省第二。招商引资累计履约项目1309个，到位资金2200亿元，比“十一五”增长129.8%。

　　——民生保障更加有力。累计投入民生资金180.38亿元，较“十一五”增加114.08亿元，年均增长13.3%。城镇登记失业率持续控制在3.7%以内，城镇新增就业8.9万人。在全省率先实现新型农村和城镇居民社会养老保险市级统筹，养老、医疗社会保险待遇高于全省平均水平。开工建设保障性住房3.3万套、竣工2.1万套，搬迁安置采煤沉陷区居民2074户，住房保障覆盖率达26.6%。初步建成区域教育、文化、医疗卫生高地，基础教育保持全省先进水平，普通高考成绩连续七年大幅提升；公共文化服务在全省实现“四个率先”，文化产业快速发展；建成省级以上临床重点专科11个，市中心医院被确定为攀西区域医疗卫生中心领头医院。全力打造“平安攀枝花”升级版，全面完成“六五”普法，群众安全感测评满意率年均达93.7%。妥善处置“8·29”煤矿事故，安全生产形势稳定好转。17个民族乡镇地区的生产总值从27.2亿元增加到51.8亿元，年均增长13%。农村贫困人口从85732人减少到19985人，贫困发生率从16.8%降至3.8%。

　　刚刚过去的2015年，是“十二五”规划的收官之年。地区生产总值比上年（下同）增长8.1%，规模以上工业增加值增长9%，全社会固定资产投资增长4.5%，社会消费品零售总额增长11.8%，一般公共预算收入下降15.2%，城镇居民人均可支配收入增长8.5%，农村居民人均可支配收入增长9.3%，单位生产总值能耗预计下降8%。一年来，我们重点抓了八个方面的工作：

　　一是首抓项目稳定社会投资。建立完善PPP项目库，储备项目43个，其中4个进入国家项目库。金沙水电站、中国重汽矿用车生产线、观音岩引水、马鞍山水库、梅子箐水库（扩建）等重大项目加快推进，攀钢钒950生产线技改、桐子林水电站、大面山风电一期、万家山光伏发电等项目竣工投产，银江水电站正式签约，毅德城、碧桂园等项目落户我市。福建金飞鱼、上海四极、天津致远、湖南红宇等知名企业与我市企业实现战略合作。全年新签约项目294个，其中投资额5亿元以上项目46个。

　　二是狠抓产业力促转型升级。在钢铁、矿业、煤炭等传统产业深受市场冲击的情况下，机械制造产业逆势增长12.9%，钒钛特色产品进入军工等高端领域，太阳能、风电等新兴产业加速壮大，全省首个农风光互补项目在我市建成，工业多点支撑格局逐步形成。跻身全国首批区域工业绿色转型发展试点城市，钒钛高新区成功创建为全国金属材料领域唯一的国家级高新区，高新技术产业产值增长29.3%。着力做精现代特色农业，积极培育新型经营主体，突出品牌打造，加强市场营销，强化科技支撑，不断延伸产业链，成功举办首届中国（攀枝花）芒果产业发展论坛，新增芒果1.4万亩、早春蔬菜2000亩。加快发展康养旅游产业，圆满承办第六届四川国际自驾游交易博览会，被纳入中国大香格里拉旅游推广联盟，被确定为中国自驾游目的地试点城市，旅游总收入增长34.6%。制定《攀枝花市电子商务发展规划（2014－2020）》，电子商务产业园建成投用，电子商务营业额从2014年的4亿元猛增至105亿元。房产新政带动房地产市场回暖，商品房销量增长12.9%。

　　三是细化举措帮扶困难企业。进一步完善稳增长措施，在全省率先推出政府搭桥贷款政策，设立攀西战略资源开发投资基金，建立完善市级领导直接联系企业、重点支持企业“红名单”、企业首席服务员、以纳税信用换取贷款信用等制度。实施“两降一缓三补贴”援企稳岗政策，企业少缴社会保险费8000万元、缓缴2.34亿元，发放稳岗补贴8452万元。强化要素协调保障，85%以上的工业企业享受电价优惠，企业铁路运费下降20%以上。发行全省唯一一支小微企业增信集合债券，21户企业受益。积极引导企业转型发展，攀钢集团新增重轨产能45万吨，东立科技、钢城集团卓越钒业在新三板挂牌。

　　四是深化改革凝聚发展动力。努力做好攀西战略资源创新开发试验区先行先试各项工作，启动实施重大科技攻关全球公开招标项目13个，攀钢高钛型高炉渣提钛及综合利用取得突破。农村土地承包经营权确权颁证按计划推进，集体林权制度主体改革基本完成，特色农业保险扩大试点范围，米易被纳入全省首批农村产权抵押融资试点县，盐边被确定为林权抵押贷款省级试点县。在全省率先实现医联体全覆盖，成为公立医院综合改革国家联系试点城市。生态文明体制、养老保险制度并轨等改革稳步实施。加快对外大通道建设，新开通至深圳航班，丽攀高速公路攀枝花段建成通车，成昆铁路扩能改造加快推进，攀大（理）高速公路开工建设。举办攀枝花—东盟经贸合作会、攀台现代特色农业和康养产业合作交流会，与成都市、北京市西城区签订战略合作框架协议。积极推进“大众创业、万众创新”，发放小额担保贷款1.17亿元，减免税收1.39亿元，扶持875人自主创业。引进专业技术人才516名，十九冶职工曾正超成为中国获得世界技能大赛金牌第一人。全面实行“三证合一、一照一码”登记模式，新增各类市场主体10073户，实现非公有制经济增加值427亿元，增长8.5%。

　　五是优化环境打造宜居城市。“五创联动”年度攻坚项目基本完成，获得“全国文明城市提名城市”称号，取得国家森林城市创建资格，完成国家园林城市申报准备，环境空气质量优良率达98.9%。开展市区视野区环城生态景观林建设，新增公共绿地2.69万平方米。乡村环境持续改善，新建幸福美丽新村76个、新村聚居点32个，建成农村廉租房161套。加快建设彝家新寨、民族团结进步新村，边远民族地区生产生活条件明显改善。

　　六是改善民生共享发展成果。投入资金40.9亿元，98项民生工程完成93项，其余5项因规划调整等原因暂缓实施或跨年完成。全力推进扶贫攻坚，通过“五个一批”脱贫6704人。将农村低保标准从2500元/年提高到3251元/年，农村贫困人口实现应保尽保。企业退休人员养老金人均增加225元/月，资助困难群众参加基本医疗保险比率达100%，大病保险制度全面实施。免除2.1万名幼儿学前教育保教费，义务教育“三免一补”、中等职业免费教育实现全覆盖。改造农村危房449户，危旧棚户区改造货币化安置率达54%。在全国“2015地级市民生发展百强榜”中，我市名列第75位，位居全省第一。

　　七是统筹施策发展社会事业。公办和普惠性幼儿园覆盖率不断提高，义务教育基本均衡检查验收通过率在全省仅次于成都，市建筑工程学校成为全国第二批中等职业教育改革发展示范学校。公立医院全面实行药品零加成，医保实现省内定点异地及时结报，公共卫生服务项目省级考核名列全省第一。成功举办纪念攀枝花开发建设50周年系列活动，三线建设博物馆、苴却砚博物馆建成开放。网格化服务管理实现社区（村）全覆盖，法治示范创建工作全面开展，社会治理能力综合测评位居全省第一，连续两年在全省群众安全感测评中位居前三。启动创建四川省质量强市示范城市，在西南地区率先建成特种设备智能化监管平台。全面加强生产、交通、食品、药品等安全监管。稳步推进1%人口抽样调查工作。扎实开展国防建设。人防、国安、检验检疫、海关、气象、供销、档案、机关事务管理、防震减灾、地方志、邮政管理、妇女儿童、残疾人等各项工作均取得新成绩。

　　八是转变作风加强自身建设。扎实开展“三严三实”专题教育，政风行风进一步好转。加快推进政务服务窗口标准化体系建设，审批项目即办率达71%。深入推进简政放权，下放市级管理权限40项。着力打造“阳光政务”，主动依法公开行政权力事项6174项，市政府门户网站跻身“全国地级市政府网站10强”。自觉接受各界监督，办结人大代表建议134件、政协提案243件，办理12345市民热线和“市长信箱”反映事项15499件。严格执行中央“八项规定”和省委省政府“十项规定”，出台市本级“三公”经费预算、差旅费管理等制度，市本级“三公”经费支出下降17.2%。加强党风廉政建设，压实“两个责任”，强化行政监察和审计监督，查办违纪违法案件380件。

　　各位代表，受国际矿产品价格低迷、国内钢铁产能严重过剩等因素影响，地区生产总值、社会消费品零售总额、城镇居民人均可支配收入等指标未能实现“十二五”规划目标，全社会固定资产投资、一般公共预算收入、城镇居民人均可支配收入未能完成市九届人大四次会议确定的年度任务。同时，经济社会发展还存在一些不足：一是产业结构转型升级步伐不快，经济抗风险能力较弱；二是道路、市政等基础设施建设相对滞后，交通瓶颈尚未完全破解；三是城乡发展还不平衡，边远民族地区同步奔小康难度较大；四是民生事业欠账较多，社会管理、城市工作有待进一步加强。政府自身也还存在创新能力不强、执行落实不力等问题，离群众满意尚有差距。

　　但令人振奋的是，五年来，我们危中寻机，推动攀西国家级战略资源创新开发试验区成功落地，加快建设国家现代农业示范区、国家钒钛高新技术产业开发区，加快创建中国阳光康养产业发展试验区，形成了“四区驱动”新格局！我们真抓实干，保障性住房、新农村建设、社保体系、社会治理等多项工作走在全省乃至全国前列，获得全国创业先进城市、全国和谐社区建设示范城市、国家知识产权示范城市等诸多殊荣，被授予“中国智慧城市推进成就奖”，连续三届荣获“全国双拥模范城”称号，开创了改革发展新局面！我们敢闯敢试，主动对接“一带一路”、长江经济带建设等重大战略，积极争创长江上游综合改革试验区，世界级钒钛产业基地、南向开放门户等战略定位获得国家及省上的肯定和支持，奠定了下一轮开发建设的坚实基础！

　　各位代表！过去的五年，我们坚定信心不动摇、克难奋进不懈怠，在转型发展的道路上取得了来之不易的成绩！这是市委正确领导，市人大、市政协监督支持，各级各部门努力拼搏，全市人民团结奋斗的结果。在此，我谨代表市政府，向全市各族人民，向驻攀解放军、武警官兵、政法干警和民兵预备役人员，向给予政府工作大力支持的人大代表、政协委员、各民主党派、工商联、无党派人士、人民团体和社会各界人士，向各位老领导、老同志，向在攀创业发展的建设者、投资者，表示衷心的感谢并致以崇高的敬意！

　　二、布局“十三五”，推动转型发展迈上新台阶

　　“十三五”时期，是我市全面小康的决胜期、结构调整的加速期、城市转型的关键期和全面改革的攻坚期。面对经济下行压力加大、区域竞争日趋激烈、结构调整持续阵痛、民生兜底任务繁重等多重挑战，我们必须传承“十二五”时期逆境奋发的昂扬精神，积极抢抓“一带一路”和长江经济带建设、“中国制造2025”、结构性改革等战略机遇，加快重构现代产业体系，加速城市转型步伐，加大民生改善力度，努力在新的起点上实现更快速度、更高水平、更好质量的跨越式发展。

　　根据市委《关于制定攀枝花市国民经济和社会发展第十三个五年规划的建议》，市政府编制了《攀枝花市国民经济和社会发展第十三个五年规划纲要（草案）》，已提交本次大会审议。

　　“十三五”时期的指导思想是：高举中国特色社会主义伟大旗帜，以马克思列宁主义、毛泽东思想、邓小平理论、“三个代表”重要思想、科学发展观为指导，深入贯彻习近平总书记系列重要讲话精神，紧紧围绕“四个全面”战略布局，全面贯彻“五大发展理念”，以产业升级和城市转型为主线，以提高经济发展质量和效益为中心，正确认识适应引领新常态，立足自身实际，突出特色优势，大力实施“三个加快建设”，全力抓好“四区驱动”，努力打造四川新的经济增长点，在全省高水平率先全面建成小康社会，奋力谱写中华民族伟大复兴中国梦攀枝花篇章。

　　“十三五”时期经济社会发展的主要目标是：到2020年，“中国钒钛之都”的国内外影响力进一步增强，“中国阳光花城”的发展活力进一步提升，“四川南向开放门户”的区域地位进一步巩固，基本实现“三个转变”“两个跨越”，即：由工矿基地向生态宜居城市转变，由钢铁之城向阳光花城转变，由三线建设城市向康养休闲城市转变；由资源大市向经济大市跨越，由总体小康向全面小康跨越。

　　围绕上述指导思想和总体目标，政府的主要任务是：

　　（一）以创新发展为动力，厚植发展优势。坚持把发展的基点放在创新上，大力推进科技创新、产业创新、管理创新、制度创新，以全面创新改革驱动转型升级，加快建设世界级钒钛产业基地，人均GDP保持全省领先。2018年，地区生产总值和城乡居民收入比2010年翻一番以上；2019年，在全省高水平率先全面建成小康社会。

　　（二）以协调发展为要义，筑牢底部支撑。坚持在协调共进中拓宽发展空间、增强发展后劲，加快推动产业升级，大力发展县域经济，统筹推进城乡建设，不断增强园区集聚能力，县区、园区对全市经济的支撑作用显著增强，区域发展更加协调，产业结构更加合理。“十三五”末，县区综合实力在全省排名提升，钒钛高新区建成千亿园区，城镇化率达到67%，新增城区面积10平方公里。

　　（三）以绿色发展为遵循，提升城市品质。坚持生态为先、标本兼治，积极构建生态经济、自然资源保障、生态环境、生态人居、生态文化、生态建设能力保障等六大体系，全面实现“五创联动”既定目标，创建成为全国文明城市、国家森林城市、国家园林城市、国家环保模范城市和中国阳光康养旅游城市。

　　（四）以开放发展为导向，夯实门户地位。坚持以开放包容的理念推动转型发展，深入对接“一带一路”、长江经济带建设等重大战略，积极融入“成渝西昆贵”经济圈，力争将攀西—六盘水经济区上升为国家战略，携手共建金沙江经济圈，将我市打造成为四川通往南亚东南亚的发展高地。2018年，成昆铁路扩能改造攀枝花至昆明段建成通车；“十三五”末，基本建成畅通安全高效的现代综合交通运输体系。

　　（五）以共享发展为目标，增进民生福祉。坚持以人为本、民生为重，全力推进脱贫攻坚，努力推动基本公共服务均等化，建立更加公平可持续的社会保障体系，建成区域科技、教育、文化、医疗卫生高地，群众获得感、归属感、幸福感显著增强。2018年，70个贫困村全部摘帽，贫困人口全部脱贫；“十三五”末，城乡居民收入继续高于全国平均水平。

　　三、奋战2016，确保“十三五”开好局

　　今年，是“十三五”决胜全面小康的开局之年，也是推进结构性改革的攻坚之年。综合研判，2016年可能是更为困难的一年，但机遇与挑战并存，希望与困难同在！政府工作的总体思路是：坚持以党的十八大、十八届五中全会、中央经济工作会议、中央城市工作会议、省委十届七次全会、市委九届九次全会等重要会议精神为指导，以“五大发展理念”为统领，以稳中求进为基调，以提高发展质量和效益为核心，以全面改革创新为牵引，以“项目推进年”为抓手，围绕“四区驱动”，坚定不移实施“三个加快建设”，着力抓好产业升级、城市转型、改革开放、环境生态、民生改善、脱贫攻坚等重点工作，千方百计去产能、去库存、去杠杆、降成本、补短板，确保主要经济指标高于全省平均水平，力争GDP达千亿，促进经济社会全面协调可持续发展。

　　全市经济社会发展的主要目标建议为：地区生产总值增长8%以上，其中，第一产业增长3.6%以上，第二产业增长8.5%以上，第三产业增长8.5%以上；规上工业增加值增长9%以上；一般公共预算收入增长5%以上；全社会固定资产投资增长10%以上；社会消费品零售总额增长10.5%以上；城镇常住居民人均可支配收入增长8.5%以上，农村常住居民人均可支配收入增长9%以上；城镇登记失业率控制在4.5%以内；居民消费价格指数增速控制在3.5%以内；节能减排完成省上下达任务。

　　围绕上述目标，重点抓好七个方面工作：

　　（一）以项目建设为重点，着力稳增长促转型

　　——强化项目带动作用。将2016年确定为“项目推进年”，推行经济工作项目化、项目工作责任化，开展项目建设“五抓五比”活动，实施重大项目89个，完成全社会固定资产投资708亿元。加强项目库建设管理，积极谋划一批事关转型发展和增添后劲的重大项目。健全落实项目会审、并联审批、市级统筹、一线推进等机制，统筹协调要素配置，全力推进对传统产业提升、新兴产业培育、基础设施改善具有带动作用的重大项目。争取设立PPP项目财政支持基金，推动PPP模式运用于更多基础设施建设及社会公益性项目。强化项目全程服务和跟踪督办，着力解决好项目落地“最后一公里”问题。

　　——力促工业企稳回升。开展降成本行动，对接争取直购电、钢铁行业脱困优惠电价、留地电量等政策，协调进一步降低主要工业品铁路运价，支持攀钢、钢城等企业优质资产和市水务集团上市融资，完善企业“红名单”制度，用好应急转贷、产业引导股权投资基金等政策性资金。有序化解过剩产能，支持攀钢、攀煤、钢城、十九冶等企业深化改革、降本增效，鼓励关联企业建立战略联盟，引导困难企业盘活存量、创新发展，推动钛白行业兼并整合。统筹推进传统产业升级和新兴产业培育，积极推行“风光林”“风光农”“风光水”等融合发展模式，培育壮大钒钛、机械制造等特色优势产业，努力抢占石墨烯等战略性新兴产业发展先机。强化项目支撑，开工建设银江水电站、亿丰瑞废弃低品位矿回收利用、一美能源储能材料及高能电池等项目，加快推进金沙水电站、中国重汽矿用车生产线、钛材深加工园、攀钢钒氮合金扩能、攀钢氯化钛白等续建项目，确保攀钢300MW矸石电厂、钢城集团渣钢铁高效回收加工、安宁铁钛硫钴综合回收利用等项目竣工投产。

　　——推动现代农业扩面提质增效。围绕国家现代农业示范区建设，加快转变农业发展方式，积极调整优化农业产业结构，着力打造优势特色农产品品牌，大力推进农业集成创新示范农庄建设和新型经营主体培育示范工程，鼓励锐华等农业龙头企业做大做强。推进现代农业产业基地建设向二半、高寒山区及边远民族地区延伸，加快发展烤烟、核桃、养殖、花卉苗木等有助于山区群众脱贫致富的特色产业，优化种植烤烟12万亩，新增早春蔬菜3000亩、特色水果1万亩、优质林果2万亩。加强农业科技创新，鼓励支持农林科技人员开展新品种、新技术研究，推广稻菜轮作、果蔬结合、种养循环等高效模式，提高良种覆盖率和土地产出率。鼓励发展农产品精深加工，引导农业与康养旅游业互动发展。加快建设马鞍山水库、梅子箐水库（扩建）、麻晃引水、太阳能提灌站等水利设施，新增和改善灌面5.8万亩，建设高标准农田2万亩。引导农业化学投入品减量使用，强化农产品全程监管和责任追溯，守住农产品安全责任底线。扎实开展第三次全国农业普查。

　　——促进服务业提档升级。将2016年确定为“旅游提质增效年”，引进推广先进康养模式，大力发展养老、养生、养心、商务会展等“旅游+”产品，加快推进阿署达、普达、红格、自驾车营地等项目建设，办好欢乐阳光节、四川冬季乡村文化旅游节和全国首届国际生态康养产业博览会暨城市论坛，力争论坛永久会址落户我市。加快建设省级现代服务业集聚区，培育壮大科技服务、文化创意、信息咨询、金融保险等新兴服务业，积极推进万达广场、御湖花都等重点项目，引导商业企业发展新业态。培育推广“房地产+”新模式，用好棚改货币化安置政策，推动房地产市场有序去库存。

　　（二）以改革创新为重点，着力夯平台添动力

　　——深化重点领域改革。抢抓四川省被纳入国家系统推进全面创新改革试验区机遇，制定专项改革方案，推进供给侧改革，不断提高改革针对性和实效性。做好国有企业“三供一业”分离移交及人员分流再就业工作，加快国投集团、建设集团转型步伐。抓好工矿废弃地复垦利用试点。全面完成农村土地承包经营权确权颁证，扩大农村产权抵押融资、特色农业保险、小型水利工程产权制度改革等试点，启动农村集体资产股份制改造试点和农村供销社综合改革试点。深化投融资体制改革，支持市级投融资平台与政策性金融机构、风险投资机构等合作设立产业、创业投资引导基金，鼓励民间资本发起设立产业投资基金。深化商事制度改革，探索实行电子营业执照及全程电子化登记管理。探索建立科技研发、成果转化一体化机制，推进攀枝花学院创新创业改革试点，鼓励金融机构设立科技专营支行。积极推进电力、税务、教育、文化、卫生、审计、机关后勤保障等体制改革。

　　——加快推进试验区建设。研究出台攀西战略资源创新开发试验区先行先试工作方案，充分发挥钒钛资源综合利用国家重点实验室、国家钒钛制品质量监督检验中心、钒钛资源综合利用产业技术创新联盟等平台作用，大力实施企业创新主体培育工程，加快制（修）订钒钛类制品标准，力争高温碳化低温氯化高炉渣提钛、低成本高品质海绵钛、钛及钛合金3D打印等重大科技攻关项目实现产业化突破。加大国家级钒钛高新区建设力度，设立钒钛投资基金，创建国家级高品质钛产品产业化技术国际科技合作基地。科学编制石墨资源开发规划和产业招商目录，建立石墨制备与应用工程技术研究中心。完善国家级阳光康养产业发展试验区创建路线图，力争成为全省首批养老健康服务业发展试点城市、全国首批医养结合试点示范城市。

　　——大力实施“互联网+”战略。制定“互联网+”行动计划，推动生产方式和发展模式变革。以工业化、信息化深度融合为主线，积极开发基于互联网技术、服务“中国制造2025”的特种钢坯、定制铸件等产品，加快建设中国钒钛电子交易中心，加大对两化深度融合项目及企业的扶持力度。以省级电子商务进农村综合示范市建设为契机，加快建设农村信息网络和乡村配送体系，与京东、腾讯等合作建设“攀枝花特色馆”，支持26度果园等发展壮大，设立一批乡镇农村电子商务服务站，建成县（区）级农村电商服务中心4个。以智慧旅游城市建设为载体，创新旅游营销模式，积极发展“旅游+”产业。加快建设电子商务产业园和电商快递园区，搭建互联网服务平台，推动商贸、餐饮、住宿等传统服务业转型升级。

　　（三）以开放合作为重点，着力借外力促发展

　　——创新机制引资引智。加强产业研究和项目包装推介，探索推行项目招商落地市场化运作模式。坚持领导带队招商、驻点招商、以商招商、节会招商，主动对接世界500强中国100强企业、行业龙头企业和知名商（协）会，积极参加首届全球川商返乡发展大会、中外知名企业四川行、西博会等重大招商活动，确保招商引资到位资金560亿元以上。深入开展“大众创业、万众创新”，支持社会力量组建创业俱乐部、创客空间等平台，探索设立创新创业孵化专项基金。加快建设高端智库和创新团队，健全人才引进、培养、使用机制，为科研人才参与创业创新松绑。

　　——务实推动区域合作。积极参与“一带一路”、长江经济带和孟中印缅经济走廊建设，加强与南亚东南亚国家的经贸交流合作。强化与毗邻市州的区域合作，推动攀西经济区一体化发展。深化同结对市州的友好合作，跟踪落实与成都市、北京市西城区议定的合作事项。办好第三届西三角区域开发合作交流会。巩固扩大攀台（湾）交流合作成果。推动重轨、钛白粉、弹簧钢板、耐磨铸件等拳头产品扩大出口，支持攀钢、钢城、十九冶等企业深化外经合作。

　　——着力提升区位优势。加快打造区域交通枢纽，扎实推进成昆铁路扩能改造、火车南站、攀大（理）高速公路、市综合客运中心及配套立交工程等项目，争取开工建设三堆子大桥等项目，做好攀盐（源）高速公路、攀宜（宾）沿江高速公路等项目前期工作，力争开通至上海航班，努力推动攀大（理）铁路、丽攀昭遵铁路、攀枝花至水富段航运资源开发纳入国家“十三五”专项规划。加快打造区域商贸物流中心，积极推进毅德城等项目，引导密地、攀青、新启等物流项目差异化发展。加快打造区域口岸中心，对接参与长江经济带海关区域通关一体化改革，积极筹建海关保税仓库，力争创建国家级出口工业产品质量安全示范区。

　　（四）以城乡建管为重点，着力强功能提品质

　　——加强城乡基础设施建设。贯彻落实中央、省委城市工作会议精神，不断完善城市规划体系，积极推进“多规合一”；努力做好海绵城市、地下综合管廊、城市轨道交通等项目可行性研究，力争启动前期工作。推进花城新区循环滚动开发，加快土地整理、花城商务、碧桂园等项目建设，做好新政务中心、规划展览馆、中心湖园林景观等项目前期工作。合理规划建设生活污水配套管网、生活垃圾中转站等市政设施，抓好棚户区、危旧房和老旧小区改造，按计划推进观音岩引水工程。启动建设全国重点镇，加快建设平地、格里坪、普威、渔门等省级试点镇，新建幸福美丽新村40个，改善提升县乡道130公里、村道80公里。

　　——加大生态保护建设力度。探索建立生态文明目标考核体系和生态环境损害责任终身追究制，科学划定生态红线，启动生态文明示范县（区）创建工作。加大钒钛、石墨等特色矿产资源的勘探和保护力度，避免无序开发、过度开发。加强生物多样性保护，完成国家级苏铁保护区三期工程。深入实施“蓝天、碧水、绿地、宁静、洁净”五大行动，探索环境污染治理第三方运维模式，建立节能减排先进适用技术推广部门联动机制，实施土壤污染治理与生态修复工程，加强污染综合防治，环境空气质量优良率保持在95%左右。

　　——推进城市精细化管理。紧扣节点推进“五创联动”，确保完成年度任务。推行城区网格化管理模式，完善城乡一体化数字城管体系，建立市区两级指挥平台及部门联动调处机制，构建“一刻钟社区便民服务圈”。深化市容环境整治，加强背街小巷治理，做好道路养护、街面保洁、扬尘治理等工作，确保通过国家卫生城市第二轮复审。加强城市交通管理，优化公交线网，整合停车站场资源，缓解交通拥堵、停车困难等问题。

　　（五）以改善民生为重点，着力办实事增福祉

　　——协调发展社会事业。加强学校标准化和信息化建设，加快农村薄弱学校改造和四十二中小、成都外国语学校攀枝花分校等项目建设，扩大公办和普惠性幼儿园比例，推进省级示范性高中与薄弱学校集团化协同发展。构建全民健身服务体系，力争承办3—5项大型体育赛事。打造三线建设党性教育基地，建成“渡口记忆”特色文化商业街、苴却砚文化旅游街区，提高本土艺术团体创作演出水平，推动群众文化繁荣发展。适时启动“健康攀枝花”建设，加快推进花城新区医院、市妇幼保健院异地新建等项目；加强“三医联动”，深化公立医院改革，健全医联体运行机制和分级诊疗制度。坚持计划生育基本国策，实施“全面二孩”政策。做好四川省质量强市示范城市创建工作。

　　——深入推进脱贫攻坚。大力实施“六大扶贫工程”，因地制宜推动山区和边远地区特色种养业加快发展，增强贫困地区群众造血能力。制定落实“五个一批”保障措施，建立完善扶贫绩效考核与责任追究制度，做好扶贫小额信贷工作。加大财政投入和资金整合力度，加快推进贫困地区水利、道路及通信网络建设。加强粮蔬种植、水果栽培、畜牧生产等农业实用技术培训，提升贫困地区群众就业创业能力。深入开展社会帮扶，引导各方力量共同推进脱贫攻坚。加大对城市贫困户的帮扶力度。全年完成24个贫困村“摘帽”、7317人脱贫。

　　——促进社会和谐稳定。健全保险、救助、优抚三大社会保障体系，深化养老、医疗、生育、工伤等社会保险制度改革，做好扶老救孤、助残助幼、就业创业等工作。健全基层治理体系，推动“六大特色社区”建设，深入开展“法律七进”活动。加快建设立体化社会治安防控体系，严厉打击各类违法犯罪行为，不断提升群众安全感。畅通群众诉求表达渠道，落实重要信访包案制度，依法处置各类矛盾纠纷。严格落实安全生产责任，强化监督管理，防止重特大安全事故发生。完善食品药品监管机制，严厉惩处制假售假行为，确保食品药品安全。抓好大中型水利水电工程移民搬迁安置、后期扶持及采煤沉陷区综合治理工作。深入开展民族团结进步创建活动。依法管理宗教事务。进一步做好国防动员、民兵预备役和“双拥”工作。

　　——办成一批民生实事。优化公交线路2条，实施路桥大修项目7个；新增45个行政村接入光纤宽带；开工建设城乡公办幼儿园6所、农村义务教育教学用房1.33万平方米；开工建设保障性住房4839套，其中改造棚户区危旧房4743套；新增就业1.6万人，失业人员再就业5000人，就业困难人员再就业1200人；启动实施老旧（弃管）小区配电设施改造，用3年时间完成4.5万户电表改造；完成市残疾人康复中心改扩建；新建乡镇（街道）规范化法律援助工作站12个；为910名贫困精神病患者提供医疗救助。

　　（六）以平稳运行为重点，着力防风险保运转

　　——全力增收节支。坚持依法征管，深入挖掘增收潜力，加快培育新的财源增长点，积极争取上级项目资金支持，多措并举增加可用财力总量。强化支出管理，细化预算编制，硬化预算约束，坚决压缩一般性支出，切实降低行政运行成本，全力保运转、保民生。厉行勤俭节约，从严管理机关事务，按要求完成公车改革。加强监督检查，提高财政资金使用效率。

　　——防控债务风险。完善政府债务管理制度，健全债务审批、统计分析、风险预警机制，合理控制举债规模。加强政府债券项目库建设，做好政府置换债券争取工作。积极开展债务资金使用绩效评价，综合采取预算安排、盘活资产、转贷置换、强化审计等措施，有效防范债务风险。

　　——优化金融生态。建立健全信贷需求常态化共享、贷款贴息、风险补偿等机制，探索开展贷款保证保险，提高金融财政资源配置效率。加强风险监测、预警和处置，完善银政企共同参与的金融风险联合化解和处置机制，有效防范和化解区域性金融风险。依法处置企业债务危机，严厉打击非法集资、高利贷等行为。

　　（七）以狠抓落实为重点，着力转作风树形象

　　——善谋善成，提升政府创新力。问计于基层，求教于群众，借智于专家，找准结构调整与经济转型的突破口，努力推动产业重构、二次创业。不断完善决策咨询、政策研究、调研成果转化等工作机制，努力打造学习型、创新型团队。积极开展试点示范，在破解要素难题、优化工作流程、推动项目落地等方面出新招、求实效。进一步求变求新、简政放权，以政府权力的“减法”换取市场活力的“加法”。

　　——求真务实，提升政府执行力。越是困难的时候，越要强调执行和落实。必须坚持一分部署、九分落实，对确定的目标、制定的政策、部署的任务，不折不扣落实到位，推进政府工作提质提速。加强绩效管理、督促检查、信息反馈、情况通报、责任追究等制度建设，狠抓跟踪问效，着力解决选择性执行、变通性执行等问题。

　　——依法行政，提升政府公信力。配合市人大积极稳妥推进地方立法，加快构建符合我市实际、接轨国际规则的城市法律体系。主动接受法律监督、民主监督、司法监督、社会监督和舆论监督，扎实推进决策公开、执行公开、信息公开、结果公开。加强行政道德建设，言必行、行必果，以行动验证表态，用实践兑现承诺。

　　——正风肃纪，提升政府约束力。按照“五个必须”要求，守纪律讲规矩，加强自我约束，做到令行禁止。强力整治慵懒散慢浮僵等不良风气，严肃查处不作为、慢作为、乱作为、办事不公、吃拿卡要等行为。层层压实“两个责任”，切实促进政风行风明显改善。

　　各位代表，新常态需要新作为，新作为开创新局面！让我们紧密团结在以习近平同志为总书记的党中央周围，在中共攀枝花市委的坚强领导下，团结带领全市各族人民，大力弘扬攀枝花精神，直面困难，迎难而上，二次创业，再创佳绩，为在全省高水平率先全面建成小康社会而努力奋斗！
